# Draft Whole-Genome Sequences of Three *Lactobacillus plantarum* Food Isolates

**DOI:** 10.1128/genomeA.00560-16

**Published:** 2016-06-16

**Authors:** Mónica D. Fernández Ramírez, Jos Boekhorst, Anne de Jong, Oscar P. Kuipers, Tjakko Abee, Masja N. Nierop Groot

**Affiliations:** a Laboratory of Food Microbiology, Wageningen University, Wageningen, the Netherlands; b Molecular Genetics, University of Groningen, Groningen, the Netherlands; c NIZO food research, Ede, the Netherlands; d Wageningen UR Food and Biobased Research, Wageningen, the Netherlands; e Top Institute Food and Nutrition (TIFN), Wageningen, the Netherlands

## Abstract

*Lactobacillus plantarum* is a widespread member of the *Lactobacillus* genus and frequently isolated from spoiled acidified food products. Here, we report the draft genome sequences of three *L. plantarum* food isolates.

## GENOME ANNOUNCEMENT

Lactobacilli are Gram-positive, generally nonmotile, bacteria that can be found in a diverse range of habitats (1). They are widely used in the food industry for preservation of dairy, meat, and vegetable products by producing lactic acid as the main fermentation product. Moreover, the probiotic properties (2) of some species may provide benefits to human and animal health. Besides their desired properties, lactobacilli are also associated with the spoilage of especially acidified food products such as ketchup (3) and dressing (4). *Lactobacillus plantarum* is a widespread member of the *Lactobacillus* genus and frequently isolated from spoiled food products, but it is also exploited for its probiotic traits (5) and fermentative capacity. Here, we report the draft genome sequences of three *L. plantarum* food isolates.

Three *L. plantarum* strains isolated from different food sources (6) were cultured for 18 h at 30°C in De Man-Rogosa-Sharpe broth (Merck). The DNA was extracted using the Wizard Genomic DNA purification kit (Promega) following the manufacturer’s instructions with a few modifications: the cell pellet was resuspended in 50 mM EDTA pH 8 (Merck); the lysozyme (Sigma) concentration used was 20 mg/ml, and the DNA was resuspended in elution buffer (10 mM Tris-Cl/pH 8.5, Qiagen). All centrifugation steps were performed at 16,000 × *g*.

The isolated DNA was sheared to 250- to 350-bp fragments and paired-end sequenced on an Illumina HiSeq2000 outsourced to BaseClear (Leiden, the Netherlands). CLC Genomics Workbench version 6.0.1 (http://www.clcbio.com/products/clc-genomics-workbench), SSPACE version 2.3 (7), and GapFiller version 1.1 (8) were used for assembly. The RAST server (9) was used to annotate the genomes.

### Nucleotide sequence accession numbers.

The genome sequences of the three *L. plantarum* strains have been deposited at DDBJ/EMBL/GenBank under the accession numbers listed in Table 1. The versions described in this paper are the first versions.

**TABLE 1:** Sequenced *Lactobacillus plantarum* strains and their isolation sources

Strain^a^	Source of isolation	Accession no.
FBR4	Cheese with garlic	LQHB00000000
FBR5	Dressing	LQHC00000000
FBR6	Onion ketchup	LQHD00000000

a FBR-number refers to the strain collection at Wageningen UR Food and Biobased Research.
